# Supplementary material for: Polygenic Panels Predicting the Susceptibility of Multiple Upper Aerodigestive Tract Cancer in Oral Cancer Patients
Source: J Pers Med. 2021 May 18;11(5):425. doi: 10.3390/jpm11050425 (PMC8158753; doi:10.3390/jpm11050425)
Supplement: Supplementary file 1 [file jpm-11-00425-s001.zip › Supplementary Materials.pdf]

Table S1. The variables used in the Least Absolute Shrinkage and Selection Operator (LASSO) regression analysis.

SNP	BP	Minor allele	Allele frequency (MPT) (%)	Allele frequency (SPT) (%)	Major allele	CHISQ	P	OR
Affx-15929578	38499997	A	0.03361	0.176	G	29.33	6.10E-08	0.1629
rs1229984	99318162	C	0.396	0.2364	T	21.97	2.76E-06	2.118
rs12675972	53104588	A	0.1613	0.3072	C	19.09	1.24E-05	0.4338
rs118169127	111379676	C	0.088	0.02218	T	18.92	1.36E-05	4.253
rs7110866	37033281	G	0.312	0.1786	A	18.3	1.89E-05	2.086
Affx-32319950	53085171	A	0.164	0.3061	G	18.25	1.94E-05	0.4447
rs76367287	157488600	C	0.124	0.04422	A	17.61	2.71E-05	3.06
rs4147539	99316323	-	0.16	0.07021	TTC	16.1	6.02E-05	2.523
rs77928255	99306663	-	0.164	0.07338	CT	15.92	6.60E-05	2.477
rs28914775	99311969	T	0.1613	0.07313	C	15.16	9.87E-05	2.437
rs28913921	99316949	-	0.16	0.07313	G	14.83	0.0001173	2.414
rs28914783	99310445	T	0.184	0.09014	C	14.83	0.0001174	2.276
rs1959792	25877117	T	0.2621	0.1502	C	14.57	0.0001349	2.01
rs7847271	115068533	A	0.04	0.1259	G	14.33	0.000153	0.2894
rs75330406	147481113	A	0.136	0.05973	G	13.46	0.0002433	2.478
Affx-22240004	35908789	T	0.1	0.03741	G	12.98	0.0003151	2.859
rs12346118	83353799	T	0.08468	0.1809	C	12.49	0.0004081	0.4189
rs2710848	168619519	G	0.128	0.2347	A	12.35	0.0004413	0.4787
rs9897457	7362313	C	0.148	0.2585	T	12.26	0.000462	0.4983
rs3760265	66875603	T	0.188	0.1003	C	12.2	0.000478	2.076
rs61401220	50919808	-	0.108	0.04422	T	12.05	0.0005192	2.617
rs9490776	123468809	A	0.02846	0.09694	G	11.48	0.0007031	0.2728

SNP	BP	Minor allele	Allele frequency (MPT) (%)	Allele frequency (SPT) (%)	Major allele	CHISQ	P	OR
rs7184686	78381741	A	0.108	0.04592	G	11.21	0.0008116	2.516
rs9554264	28107218	T	0.348	0.2211	C	14.72	0.0001245	1.88
rs1042026	99307309	T	0.2903	0.1803	C	12.61	0.0003845	1.86
rs11730075	99316676	A	0.2903	0.1804	G	12.52	0.0004024	1.858
rs975833	99280582	G	0.292	0.1832	C	12.24	0.0004678	1.839
rs141057871	14990529	A	0.2195	0.1241	G	12.22	0.0004732	1.984
rs10005290	99308253	A	0.288	0.1803	C	12.17	0.0004859	1.839
rs2075633	99317841	T	0.288	0.1803	C	12.17	0.0004859	1.839
rs4873692	53033610	T	0.464	0.3367	G	12.13	0.0004972	1.705
rs11819833	5592180	C	0.252	0.1514	T	11.97	0.000542	1.889
rs7129229	92367190	T	0.2358	0.1376	A	11.92	0.0005558	1.933
rs549317178	18609800	T	0.152	0.2611	-	11.83	0.0005824	0.5073
rs2066701	99317256	G	0.288	0.182	A	11.74	0.0006118	1.818
rs848	132660808	A	0.4355	0.3123	C	11.64	0.0006448	1.699
rs1471535	37073188	A	0.252	0.1531	C	11.51	0.0006938	1.864
rs6806581	23609675	G	0.132	0.233	A	11.06	0.0008801	0.5006
rs11255400	7845658	A	0.028	0.08503	G	9.001	0.002698	0.31
rs112433986	216274537	-	0.024	0.07679	A	8.526	0.003501	0.2956
rs149089400	143802452	A	0.08	0.02041	G	8.48	0.00359	4.174
rs60993826	231305687	T	0.024	0.07363	C	7.797	0.005232	0.3094
rs79454125	82271006	C	0.024	0.07313	T	7.691	0.00555	0.3117
rs145105977	85104040	C	0.024	0.07143	T	7.308	0.006866	0.3197
rs12797844	70252484	C	0.02016	0.06143	T	6.35	0.01174	0.3144
